# A Deep Residual U-Net Algorithm for Automatic Detection and Quantification of Ascites on Abdominopelvic Computed Tomography Images Acquired in the Emergency Department: Model Development and Validation

**DOI:** 10.2196/34415

**Published:** 2022-01-03

**Authors:** Hoon Ko, Jimi Huh, Kyung Won Kim, Heewon Chung, Yousun Ko, Jai Keun Kim, Jei Hee Lee, Jinseok Lee

**Affiliations:** 1 Department of Biomedical Engineering Kyung Hee University Yongin-si Republic of Korea; 2 The Department of Radiology Ajou University School of Medicine Suwon Republic of Korea; 3 Department of Radiology Asan Medical Center University of Ulsan College of Medicine Seoul Republic of Korea; 4 Research Institute of Radiology Asan Medical Center University of Ulsan College of Medicine Seoul Republic of Korea; 5 Biomedical Research Center Asan Institute for Life Sciences Asan Medical Center Seoul Republic of Korea

**Keywords:** ascites, computed tomography, deep residual U-Net, artificial intelligence

## Abstract

**Background:**

Detection and quantification of intra-abdominal free fluid (ie, ascites) on computed tomography (CT) images are essential processes for finding emergent or urgent conditions in patients. In an emergency department, automatic detection and quantification of ascites will be beneficial.

**Objective:**

We aimed to develop an artificial intelligence (AI) algorithm for the automatic detection and quantification of ascites simultaneously using a single deep learning model (DLM).

**Methods:**

We developed 2D DLMs based on deep residual U-Net, U-Net, bidirectional U-Net, and recurrent residual U-Net (R2U-Net) algorithms to segment areas of ascites on abdominopelvic CT images. Based on segmentation results, the DLMs detected ascites by classifying CT images into ascites images and nonascites images. The AI algorithms were trained using 6337 CT images from 160 subjects (80 with ascites and 80 without ascites) and tested using 1635 CT images from 40 subjects (20 with ascites and 20 without ascites). The performance of the AI algorithms was evaluated for diagnostic accuracy of ascites detection and for segmentation accuracy of ascites areas. Of these DLMs, we proposed an AI algorithm with the best performance.

**Results:**

The segmentation accuracy was the highest for the deep residual U-Net model with a mean intersection over union (mIoU) value of 0.87, followed by U-Net, bidirectional U-Net, and R2U-Net models (mIoU values of 0.80, 0.77, and 0.67, respectively). The detection accuracy was the highest for the deep residual U-Net model (0.96), followed by U-Net, bidirectional U-Net, and R2U-Net models (0.90, 0.88, and 0.82, respectively). The deep residual U-Net model also achieved high sensitivity (0.96) and high specificity (0.96).

**Conclusions:**

We propose a deep residual U-Net–based AI algorithm for automatic detection and quantification of ascites on abdominopelvic CT scans, which provides excellent performance.

## Introduction

Currently, computed tomography (CT) of the abdomen and pelvis continues to be the primary modality for patients who visit an emergency department for abdominal pain or trauma, especially in time-critical situations [1]. In emergency situations, immediate assessment of CT is required, but limited radiologic resources may hamper or delay the recognition of patients who need urgent intervention or surgery [2]. To overcome these challenges, the development of artificial intelligence (AI) techniques using a deep learning model (DLM) to detect critical findings on CT images might be a possible solution [3].

On abdominopelvic CT images, several findings indicate emergent or urgent conditions, including ascites (ie, intra-abdominal free fluid), free gas, abscess, and fat stranding [1]. Of these, presence of ascites is a common finding in various acute abdominal diseases and intra-abdominal organ injury [4]. In addition, quantification of ascites is also important, as the amount of free fluid may correlate with the severity of injury [5].

There has been only one study that developed a DLM to detect ascites, but that DLM did not quantify the amount of fluid. That study used a convolutional neural network (CNN) classification algorithm to discriminate CT images with fluid from CT images without fluid, which achieved 85% sensitivity and 95% specificity [3]. In contrast to that study, we attempted to develop an AI segmentation algorithm that can perform both detection of ascites as well as the quantification of the volume of ascites at the same time. A segmentation value of zero means no ascites, and segmentation values of the area of ascites can be used to quantify the exact volume of ascites. In addition, we tried to increase the detection accuracy of the AI algorithm.

Recently, several state-of-the-art DLM algorithms for segmentation of CT images have been proposed, including U-Net [6], bidirectional U-Net [7], recurrent residual U-Net (R2U-Net) [8], and a deep residual U-Net CNN [9]. U-Net is one of the deep learning networks with an encoder-decoder architecture, which employs skip connections to combine low-level feature maps from an encoder and high-level semantic feature maps from a decoder. Since U-Net allows for the use of location and context at the same time, and works well with very few training samples, it has been widely used in medical image segmentation [10-13]. In addition, variant models based on U-Net, such as bidirectional U-Net, R2U-Net, and a deep residual U-Net, have been applied to medical image segmentation.

Of these, we hypothesized that a deep residual U-Net might be the best algorithm for segmentation because it combines the strengths of residual learning and U-Net. The residual network has several advantages [14-16]. First, it accelerates the speed of training of the deep networks. Second, it requires fewer parameters by increasing the depth of the network instead of widening the network. Third, it reduces the effect of the vanishing gradient problem. Last, it provides high accuracy in network performance, especially in image classification and segmentation. However, no study has been reported that used a deep residual U-Net algorithm for the segmentation of ascites on CT images. Thus, we aimed to develop an optimized deep residual U-Net algorithm to detect and quantify ascites on CT images, along with a performance comparison with other state-of-the-art networks.

## Methods

### Patients

This study was approved by the institutional review board of Ajou University Hospital. Informed consent was waived. From January 1 to March 1, 2020, a total of 1055 patients visited the emergency department and had abdominopelvic CT scans performed. Of these, 205 patients had ascites detected on their CT images. After excluding 5 patients who underwent noncontrast CT only, we included 200 patients as the ascites group. Of the remaining 850 patients without ascites, we chose 200 age- and sex-matched controls using the MatchIt package (version 4.0.0) in R software (version 4.0.2; The R Foundation). From the patients in the ascites group and the control group, we randomly selected 100 patients with ascites and 100 patients without ascites for training and testing AI models.

The clinical characteristics of the patients in the control group and ascites group are summarized in Table 1. In the control group, out of 200 patients, unknown cause of abdominal pain (n=140, 70.0%) was the most common disease category with normal abdominopelvic CT. In contrast, in the ascites group, out of 200 patients, cancer (n=42, 21.0%), liver cirrhosis (n=52, 26.0%), blunt trauma (n=37, 18.5%), and infection (n=28, 14.0%) were the main causes for emergency department visits. The majority of ascites were identified in the pelvic cavity.

**Table 1:** Demographic and clinical data of participants in the control group and ascites group.

Variables			Control group (n=200)	Ascites group (n=200)
**Demographics**				
	**Sex, n (%)**			
		Female	92 (46.0)	101 (50.5)
		Male	108 (54.0)	99 (49.5)
	Age in years, mean (SD)		59.7 (13.8)	60.2 (15.3)
**Amount of ascites, n (%)**				
	Large		0 (0)	92 (46.0)
	Moderate		0 (0)	47 (23.5)
	Small		0 (0)	61 (30.5)
**Disease category, n (%)**				
	Cancer		14 (7.0)	42 (21.0)
	Congestive heart failure		0 (0)	3 (1.5)
	Liver cirrhosis		1 (0.5)	51 (25.5)
	Acute liver failure		0 (0)	3 (1.5)
	Infection		7 (3.5)	28 (14.0)
	Blunt trauma		5 (2.5)	37 (18.5)
	Postoperative status		32 (16.0)	5 (2.5)
	Intestinal obstruction		1 (0.5)	10 (5.0)
	Renal failure		0 (0)	10 (5.0)
	Unknown cause of abdominal pain		140 (70.0)	11 (5.5)

### CT Image Acquisition and Analysis

All patients underwent abdominopelvic CT scans using multichannel multidetector scanners (Somatom Definition Edge or Somatom Definition AS, Siemens Healthineers). Contrast-enhanced CT scans were obtained with intravenous injections of 100 to 150 mL of a nonionic contrast medium (Iopamiro 300, Bracco Imaging; Omnipaque 300, GE Healthcare) at a rate of 2.5 to 3 mL/s. The scan parameters were as follows: beam collimation, 0.75 mm; slice thickness, 5 mm; effective tube current–time charge, 200 to 260 mAs; and voltage, 100 to 120 kVp. In this study, we used only contrast-enhancement CT images. If there were multiphasic CT images, we chose portal venous phase CT images for AI training and validation.

An expert abdominal radiologist (JH, with 13 years’ experience) selected CT slices that demonstrated ascites from the ascites group (2461 images from 100 patients). Then, the radiologist selected corresponding CT slices from the control group (5511 images from 100 patients). The radiologist created segmentation maps of ascites in the selected CT slices using ImageJ software (version 1.53j; National Institutes of Health), which served as ground-truth labels.

### Training and Validation Data Set and Augmentation

Table 2 summarizes the training and testing data sets, which were randomly split with a ratio of 8:2 into a training set and a testing set, respectively, in a stratified fashion. The testing set was used only for an independent test of developed models and was never used for training and internal validation.

The training data set was then further separated for training the model (80% of the training set) and for internal validation (20% of the training set). To balance the two groups’ images as well as reduce overfitting on training data, we employed image augmentation. We randomly drew the training images and applied them to the random combination of angle rotation between –10 and 10 degrees and vertical and horizontal flip. Finally, a total of 48,874 CT images were augmented: 24,437 images from patients with ascites and 24,437 images from healthy subjects.

**Table 2:** Summary of training and testing data sets.

Group	Training data, n (%)			Testing data, n (%)			Total, n (%)	
	Subjects (n=160)	Images (n=6337)	Subjects (n=40)		Images (n=1635)	Subjects (n=200)		Images (n=7972)
Ascites	80 (50.0)	1969 (31.1)	20 (50.0)		492 (30.1)	100 (50.0)		2461 (30.9)
Control	80 (50.0)	4368 (68.9)	20 (50.0)		1143 (69.9)	100 (50.0)		5511 (69.1)

### Preprocessing

For all of the images in the training and testing data sets, we first set the abdomen window according to the Digital Imaging and Communications in Medicine (DICOM) standard, which is a 400 Hounsfield Unit (HU) window width and a 60 HU window level. Subsequently, we down-sampled the DICOM images as well as masked images from an image size of 512 × 512 pixels to 256 × 256 pixels, and we normalized the pixel values to a range between 0 and 1.

### Deep Residual U-Net

**Figure 1:**
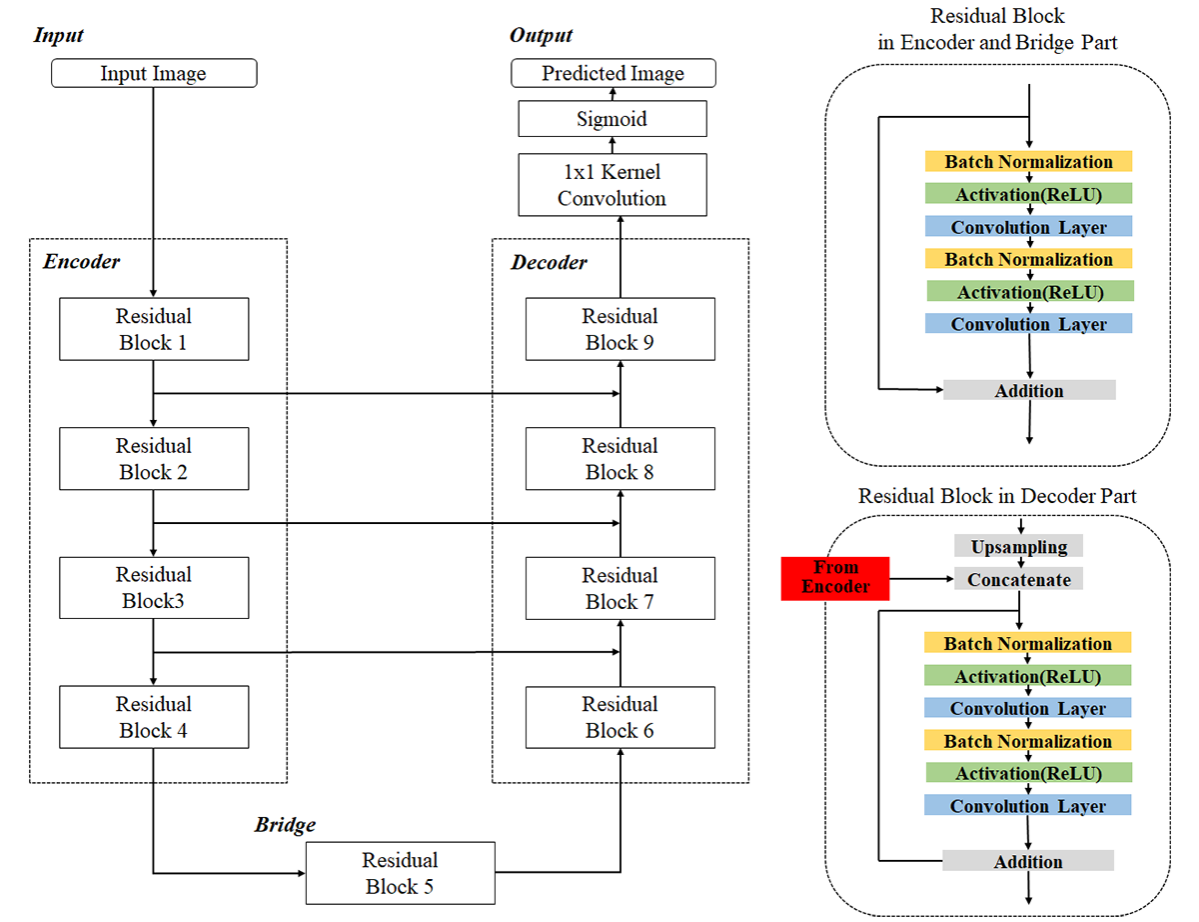
The architecture of our proposed model for ascites region segmentation based on a single abdomen computed tomography (CT) image. ReLU: rectified linear unit.

We proposed the model for ascites region segmentation based on a single abdomen CT image using a deep residual U-Net algorithm. Figure 1 shows the architecture of our proposed model, which is comprised of three parts: an encoder, a bridge, and a decoder. In the encoder part, the normalized 256 × 256–pixel image as input is encoded into a denser representation. The decoding part, on the other hand, recovers the ascites region by pixel-wise categorization. The bridge part connects the encoding and decoding parts.

In this study, we used the residual learning approach to facilitate the training of deep neural networks and take advantage of the ascites segmentation performance gain in abdomen CT images. Each residual block consists of two paths. One path is the forward pass through batch normalization, activation, and convolutional layers, which are repeated twice. The other path is the skip connection. The outputs from the two paths are added as a single output. In the encoder part (ie, residual blocks 1-4), the output from the residual block is fed into both a subsequent residual block and one of the residual blocks in the decoder part (ie, residual blocks 6-9). Thus, in the decoder part, the residual block has two inputs: one from the encoder and the other from the previous residual block output. In the bridge part, another residual block (ie, residual block 5) connects the encoding part to the decoding part. In this study, we found that four residual blocks in each of the encoder and decoder parts provided the best performance in ascites segmentation. We describe our numerical results and comparisons in the Results section. For all residual blocks, we used the rectified linear unit activation function.

Table 3 summarizes the hyperparameters of the convolutional layers and the output size in each residual block. The normalized 256 × 256 × 3–pixel image as input was fed into residual block 1, where we used the two convolutional layers with 32 3 × 3–pixel kernels and a stride of 1 with zero padding. The activation map with a size of 256 × 256 × 32 pixels from residual block 1 was fed into both residual block 2 and residual block 9. In residual block 2, we used two convolutional layers with 64 3 × 3–pixel kernels and strides of 2 and 1 with zero padding. The activation map with a size of 128 × 128 × 64 pixels from residual block 2 was fed into both residual block 3 and residual block 8. In residual block 3, we used two convolutional layers with 128 3 × 3–pixel kernels and strides of 2 and 1 with zero padding. The activation map with a size of 64 × 64 × 128 pixels from residual block 3 was fed into both residual block 4 and residual block 7. In residual block 4, we used two convolutional layers with 256 3 × 3–pixel kernels and strides of 2 and 1 with zero padding. The activation map with a size of 32 × 32 × 256 pixels from residual block 4 was fed into residual block 5, where we used two convolutional layers with 512 3 × 3–pixel kernels and strides of 2 and 1 with zero padding.

The activation map with a size of 16 × 16 × 512 pixels from residual block 5 was fed into residual block 6, where the input was first up-sampled to 32 × 32 × 512 pixels. In residual block 6, we used two convolutional layers with 256 3 × 3–pixel kernels and a stride of 1 with zero padding. The activation map with a size of 32 × 32 × 256 pixels from residual block 6 was fed into residual block 7, and it was concatenated with the output from residual block 3. When the two inputs were concatenated, the output from residual block 6 was up-sampled to match the size. In residual block 7, we used two convolutional layers with 128 3 × 3–pixel kernels and a stride of 1 with zero padding. The activation map with a size of 64 × 64 × 128 pixels from residual block 7 was fed into residual block 8, and it was up-sampled and concatenated with the output from residual block 2. In residual block 8, we used two convolutional layers with 64 3 × 3–pixel kernels and a stride of 1 with zero padding. The activation map with a size of 128 × 128 × 64 pixels from residual block 8 was fed into residual block 9, and it was up-sampled and concatenated with the output from residual block 1. In residual block 9, we used two convolutional layers with 32 3 × 3–pixel kernels and a stride of 1 with zero padding.

The activation map with a size of 256 × 256 × 32 pixels was then fed into the convolutional layer with a single 1 × 1–pixel kernel and a stride of 1. The resultant activation map with a size of 256 × 256 × 1 pixels was finally fed into a sigmoid layer, which provided the pixel-wise probability of the presence or absence of ascites.

**Table 3:** Hyperparameters of convolutional layers according to each layer and unit level.

Model part, unit level, and layer				Kernel				Strides, n		Output size, pixels
				Filter size, pixels		Filters, n				
**Input**										
	N/A^a^			N/A		N/A		N/A		256 × 256 × 3
**Encoder**										
	**Residual block 1**									
		Convolutional layer 1	3 × 3		32		1		256 × 256 × 32	
		Convolutional layer 2	3 × 3		32		1		256 × 256 × 32	
	**Residual block 2**									
		Convolutional layer 3	3 × 3		64		2		128 × 128 × 64	
		Convolutional layer 4	3 × 3		64		1		128 × 128 × 64	
	**Residual block 3**									
		Convolutional layer 5	3 × 3		128		2		64 × 64 × 128	
		Convolutional layer 6	3 × 3		128		1		64 × 64 × 128	
	**Residual block 4**									
		Convolutional layer 7	3 × 3		256		2		32 × 32 × 256	
		Convolutional layer 8	3 × 3		256		1		32 × 32 × 256	
**Bridge**										
	**Residual block 5**									
		Convolutional layer 9	3 × 3		512		2		16 × 16 × 512	
		Convolutional layer 10	3 × 3		512		1		16 × 16 × 512	
**Decoder**										
	**Residual block 6**									
		Convolutional layer 11	3 × 3		256		1		32 × 32 × 256	
		Convolutional layer 12	3 × 3		256		1		32 × 32 × 256	
	**Residual block 7**									
		Convolutional layer 13	3 × 3		128		1		64 × 64 × 128	
		Convolutional layer 14	3 × 3		128		1		64 × 64 × 128	
	**Residual block 8**									
		Convolutional layer 15	3 × 3		64		1		128 × 128 × 64	
		Convolutional layer 16	3 × 3		64		1		128 × 128 × 64	
	**Residual block 9**									
		Convolutional layer 17	3 × 3		32		1		256 × 256 × 32	
		Convolutional layer 18	3 × 3		32		1		256 × 256 × 32	
**Output**										
	**N/A**									
		Convolutional layer 19	1 × 1		1		1		256 × 256 × 1	
	**N/A**									
		Sigmoid layer	N/A		N/A		N/A		256 × 256 × 1	

^a^N/A: not applicable; this model part did not include this parameter.

### Implementation

We implemented our proposed model using the TensorFlow package (version 1.14.0), which provides a Python (version 3.6.8; Python Software Foundation) application programming interface for tensor manipulation. We also used Keras (version 2.2.4) as the official front end of TensorFlow. We trained the models with the Adam optimizer with a learning rate of 0.0001, a batch size of 16, and the loss functions of binary cross-entropy and dice loss [17] on the GeForce GTX 1080 Ti GPU (NVIDIA Corporation).

For the performance evaluation, 5-fold cross-validation was performed to confirm its generalization ability. The augmented training data set (n=48,874) was randomly shuffled and divided into five equal groups in a stratified manner. Subsequently, four groups were selected for training the model, and the remaining group was used for validation. This process was repeated five times by shifting the internal validation group. Then, we averaged the mean validation costs of the five internal validation groups according to each epoch and found the optimal epoch that provides the lowest validation cost. The testing data set was evaluated only after the model was completely trained using the training and validation data set.

### Performance Evaluation

We first investigated the effect of the number of residual blocks. For the comparison, we repeated the same procedure of the 5-fold cross-validation for two to five residual blocks. For further performance comparison, we compared our proposed method with U-Net [6], bidirectional U-Net [7], and R2U-Net [8].

For the segmentation evaluation, we quantized the mean intersection over union (mIoU), which is defined as the size of the intersection divided by the size of the union. Particularly for the nonascites images, no pixel was segmented, as we quantized the value by zero. If there were no segmentation results for the nonascites image, we quantized the value by 1.

In addition to the segmentation performance, we evaluated the detection performance. If the mIoU value was equal or greater than a certain threshold value, we declared it by ascites image. For the detection performance, we plotted a receiver operating characteristic (ROC) curve and calculated the area under the ROC curve (AUROC). Subsequently, we also evaluated the sensitivity, specificity, accuracy, balanced accuracy, precision, and F1 score. More specifically, we calculated true positives (TPs), false positives (FPs), true negatives (TNs), and false negatives (FNs) and computed the following metrics:

Sensitivity = TP / (TP + FN) **(1)**

Specificity = TN / (TN + FP) **(2)**

Accuracy = (TP + TN) / (TP + TN + FP + FN) **(3)**

Balanced Accuracy = (Sensitivity + Specificity) / 2 **(4)**

Precision = TP / (TP + FP) **(5)**

F1 score = 2 × (Sensitivity × Precision) / (Sensitivity + Precision) **(6)**

where TP is the amount of ascites data correctly classified as ascites, TN is the amount of nonascites data correctly classified as normal, FP is the amount of nonascites data misclassified as ascites, and FN is the amount of ascites data misclassified as normal. Two abdominal radiologists (JH and KWK) also evaluated the factors influencing the performance of detection and segmentation of ascites through a systematic review of all original CT images and AI results of the testing data set.

## Results

### Performance in the Cross-Validation

Table 4 summarizes the cross-validation results of various AI models for ascites segmentation performance and ascites detection accuracy using mIoU and AUROC, respectively. Deep residual U-Net models with various numbers of residual blocks generally provided higher mIoU and AUROC values than any other state-of-the-art methods [6-8]. Among the deep residual U-Net models with various numbers of residual blocks, the model with four residual blocks provided the highest mIoU (0.87) for the segmentation performance and the highest AUROC (0.99) for the detection performance. The computational time for training for the deep residual U-Net model with four residual blocks and 5-fold cross-validation was 27 hours. The overall computational time for testing was 30 minutes.

**Table 4:** Cross-validation results for the training data set comparing the mIoU for segmentation performance and AUROC for detection across models.

Model	mIoU^a^ (SD)	AUROC^b^ (SD)
Deep residual U-Net (two residual blocks)	0.86 (0.03)	0.97 (0.02)
Deep residual U-Net (three residual blocks)	0.86 (0.02)	0.98 (0.01)
Deep residual U-Net (four residual blocks)	0.87 (0.02)	0.99 (0.01)
Deep residual U-Net (five residual blocks)	0.69 (0.46)	0.69 (0.01)
U-Net [6]	0.84 (0.02)	0.96 (0.01)
Bidirectional U-Net [7]	0.82 (0.01)	0.91 (0.01)
Recurrent residual U-Net [8]	0.74 (0.02)	0.90 (0.01)

^a^mIoU: mean intersection over union; this is an index of the segmentation performance.

^b^AUROC: area under the receiver operating characteristic curve; this is an index of detection accuracy.

We also investigated the effect of the number of convolutional layers in each residual block. Table 5 summarizes the cross-validation results when the number of convolutional layers changes from two to four. It shows that the deep residual U-Net model with the two convolutional layers in each residual block provided the highest values of mIoU (0.87) and AUROC (0.99), followed by three convolutional layers (mIoU=0.83 and AUROC=0.98) and four convolutional layers (mIoU=0.69 and AUROC=0.69).

**Table 5:** Effect of the number of convolutional layers in each residual block on cross-validation results with the training data set.

Model	mIoU^a^ (SD)	AUROC^b^ (SD)
Deep residual U-Net with two convolutional layers in each residual block	0.87 (0.02)	0.99 (0.01)
Deep residual U-Net with three convolutional layers in each residual block	0.83 (0.03)	0.98 (0.02)
Deep residual U-Net with four convolutional layers in each residual block	0.69 (0.02)	0.69 (0.01)

^a^mIoU: mean intersection over union; this is an index of the segmentation performance.

^b^AUROC: area under the receiver operating characteristic curve; this is an index of the detection accuracy.

### Performance With the Testing Data Set

Table 6 summarizes the testing data results for segmentation performance using mIoU and detection accuracy using AUROC when the number of convolutional layers changes from two to four. Similar to the cross-validation results, these results also show that the deep residual U-Net model with four residual blocks including two convolutional layers provided the highest mIoU (0.87) and AUROC (0.96) with the isolated testing data set (n=1635).

With the two convolutional layers in each residual block, we also evaluated and compared the segmentation and detection performances. For the performance comparison, we changed the number of residual blocks from two to five and tested each model using the testing data set. Also, we tested with U-Net, bidirectional U-Net, and R2U-Net. Table 7 summarizes the performance comparison. The results also show that the deep residual U-Net with four residual blocks provided the highest mIoU and AUROC values. We also note that the deep residual U-Net with three residual blocks also provided high values of mIoU and AUROC, which were higher than any other state-of-the-art methods, indicating that the deep residual U-Net approach was more appropriate for the ascites segmentation and detection.

**Figure 2:**
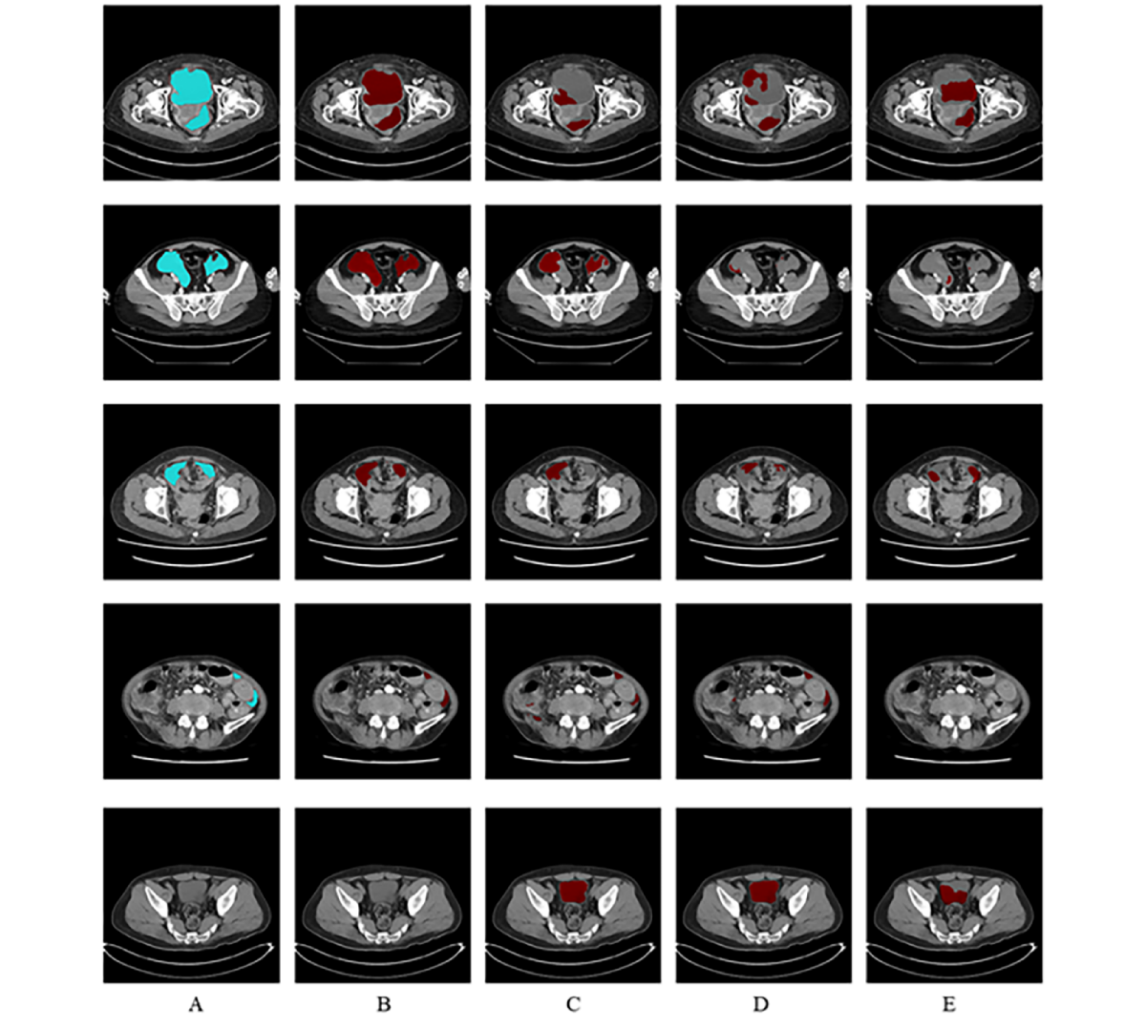
Five examples of ascites segmentation results using each model. A. The original computed tomography (CT) images and the ground-truth masking images. B. Our proposed model. C. The U-Net model. D. The bidirectional U-Net model. E. The recurrent residual U-Net model. Each row represents a different example of CT images. Blue represents the ground-truth masking images, and red represents the resultant segmented images.

The representative images of ascites segmentation are presented in Figure 2. The left-hand column (A) includes the original CT images and the ground-truth masking images. Five examples of ascites segmentation results are shown using our proposed model (B) and comparing them with those using U-Net (C), bidirectional U-Net (D), and R2U-Net (E). Our proposed model correctly segmented the ascites region regardless of its pattern and size (the top four panels in column B). In addition, for the nonascites images, the segmentation results were not shown (the bottom panel in column B).

Table 8 summarizes the testing data results of detection accuracy with the metrics of sensitivity, specificity, accuracy, balanced accuracy, precision, and F1 score. The deep residual U-Net with four residual blocks provided the highest accuracy metrics: sensitivity=0.96, specificity=0.96, accuracy=0.96, balanced accuracy=0.96, precision=0.91, and F1 score=0.93. Based on these results, we proposed our deep residual U-Net with four residual blocks as an optimal AI algorithm for automatic ascites detection and segmentation on abdominopelvic CT scans.

**Table 6:** Effect of the number of convolutional layers in each residual block on the testing data set results for the deep residual U-Net model with four residual blocks.

Model	mIoU^a^ (SD)	AUROC^b^
Deep residual U-Net with two convolutional layers in each residual block	0.87 (0.26)	0.96
Deep residual U-Net with three convolutional layers in each residual block	0.84 (0.27)	0.94
Deep residual U-Net with four convolutional layers in each residual block	0.74 (0.31)	0.72

^a^mIoU: mean intersection over union; this is an index of the segmentation performance.

^b^AUROC: area under the receiver operating characteristic curve; this is an index of the detection accuracy.

**Table 7:** Segmentation performance and detection accuracy of artificial intelligence models with the testing data set.

Model	mIoU^a^ (SD)	AUROC^b^
Deep residual U-Net (two residual blocks)	0.81 (0.33)	0.87
Deep residual U-Net (three residual blocks)	0.86 (0.28)	0.93
Deep residual U-Net (four residual blocks)	0.87 (0.26)	0.96
Deep residual U-Net (five residual blocks)	0.70 (0.46)	0.70
U-Net [6]	0.80 (0.33)	0.90
Bidirectional U-Net [7]	0.77 (0.35)	0.86
Recurrent residual U-Net [8]	0.67 (0.41)	0.81

^a^mIoU: mean intersection over union; this is an index of the segmentation performance.

^b^AUROC: area under the receiver operating characteristic curve; this is an index of the detection accuracy.

**Table 8:** Detection performance metrics of artificial intelligence models with the testing data set.

Model	Sensitivity	Specificity	Accuracy	Balanced accuracy	Precision	F1 score
U-Net [6]	0.92	0.90	0.90	0.91	0.79	0.85
Bidirectional U-Net [7]	0.94	0.86	0.88	0.90	0.74	0.83
Recurrent residual U-Net [8]	0.85	0.81	0.82	0.83	0.66	0.74
Deep residual U-Net (four residual blocks)	0.96	0.96	0.96	0.96	0.91	0.93

### Factors Influencing the Performance

**Figure 3:**
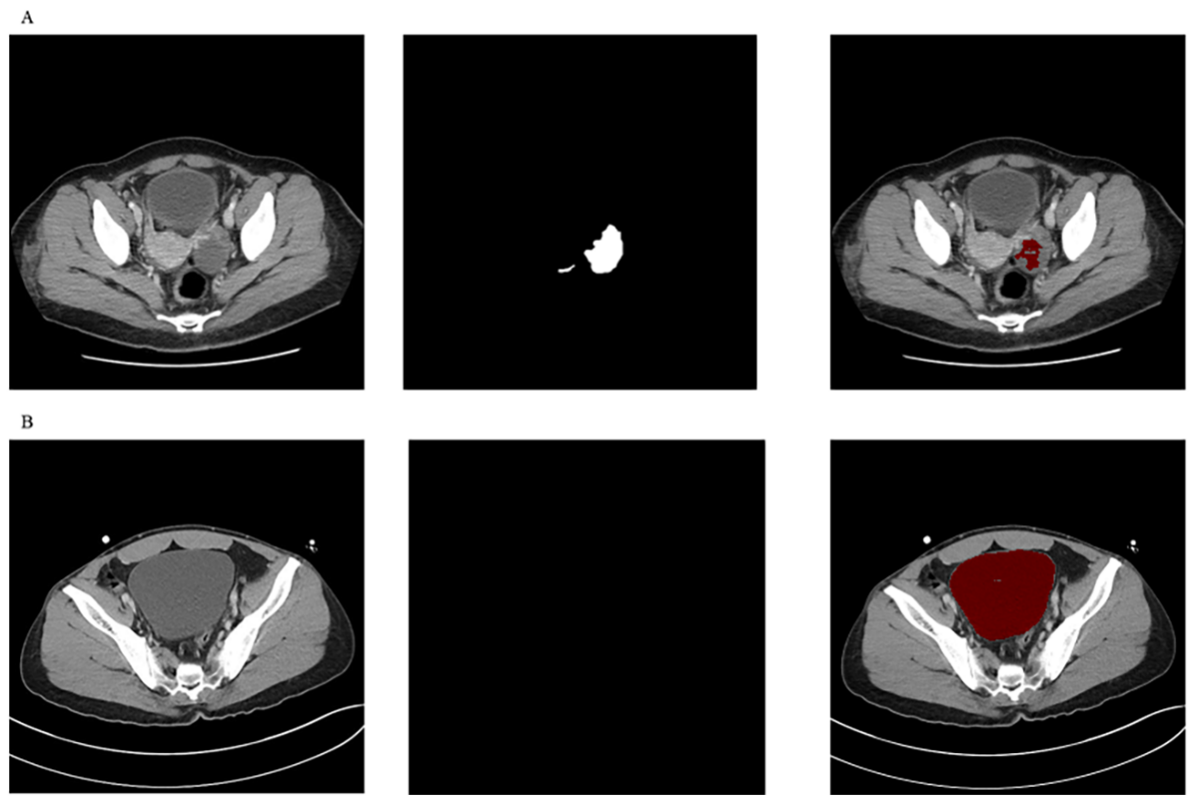
Examples of incorrect segmentation results. The left-hand column includes the original computed tomography (CT) images, the middle column includes the ground-truth masking images, and the right-hand column includes the segmented results by our deep residual U-Net algorithm. A. In a patient with a left ovarian cyst, our artificial intelligence (AI) algorithm detected fluid within the ovarian cyst as ascites. B. In a patient with a fully distended bladder, our AI algorithm detected fluid in the bladder as ascites. Red represents the resultant segmented images.

Through the expert review of all images in the testing data set by two radiologists (JH and KWK), there were two categories of false positive images. The AI algorithm could not differentiate between ovarian cysts of a substantial size (>3 cm in diameter) and ascites (Figure 3A). In contrast, normal physiologic ovarian cysts were correctly identified by our algorithm. The AI algorithm could not differentiate ascites from a fully distended urinary bladder (Figure 3B). However, the AI algorithm was able to differentiate ascites from a partially distended or collapsed urinary bladder.

All the false negative images showed a small amount of ascites. Two radiologists determined that all the false negative results were clinically insignificant.

## Discussion

### Principal Findings

In this study, for the first time, we developed a deep residual U-Net model for the segmentation of ascites on CT images, which provided higher accuracy compared with state-of-the-art networks, including U-Net, bidirectional U-Net, and R2U-Net. Our study results demonstrated that our AI algorithm was able to detect and quantify ascites in the abdominopelvic cavity. Our proposed algorithm was the deep residual U-Net model, which achieved 96% sensitivity, 96% specificity, and 96% accuracy for ascites detection with the testing data set. The segmentation performance was also high, with an mIoU of 0.87, when comparing the AI segmentation results and ground-truth values. However, the ground-truth values were generated by a human expert, and human error may have affected the drawing of the ascites boundaries. Thus, we believe that the AI segmentation algorithm might be more accurate for drawing the boundary areas of ascites in general.

The deep residual U-Net model outperformed the state-of-the-art algorithms, including U-Net, bidirectional U-Net, and R2U-Net. The deep residual U-Net model combined the strengths of residual learning and U-Net architecture [9]. The network was built with residual units and has similar architecture to that of U-Net. The benefits of this model are three-fold: (1) residual units facilitate the training of deep networks, (2) the vanishing gradient problem is reduced, and (3) the rich skip connections within the network could facilitate information propagation, resulting in higher mIoU values. Integration of the residual network with standard U-Net architecture enabled us to extract robust discriminative features from input CT images.

In general, the concept of U-Net is to stitch low-level features into corresponding high-level features, thereby adding low-level texture features to high-level semantic features. Thus, U-Net with a deep layer can provide better segmentation results. However, an excessive increase in the number of network layers tends to decrease segmentation accuracy. This issue can be solved by adding a residual unit to U-Net, which can make use of the merits of the residual network [6]. A deep residual U-Net model has been used for lung segmentation in CT scans [9], joint segmentation in CT scans [18], and vulnerable plaque segmentation in optical coherence tomography images [19]. These prior studies consistently reported the high segmentation performance of a deep residual U-Net model. In addition, our proposed deep residual U-Net model has an advantage over other U-Net models, in that it requires fewer parameters compared to other tree models [6-8]. Table 9 summarizes the comparison of the number of parameters for each model. Our proposed model includes 18,855,137 weights and biases, which represents only 54.5% of the parameters from U-Net. Also, this represents only 34.0% and 78.1% of the parameters from bidirectional U-Net and R2U-Net, respectively.

**Table 9:** Comparison of the number of parameters for each U-Net model.

Model	Trainable parameters, n	Nontrainable parameters, n	Total parameters, n
Our proposed model	18,840,545	14,592	18,855,137
U-Net [6]	34,600,353	14,016	34,614,369
Bidirectional U-Net [7]	55,398,798	1408	55,400,197
Recurrent residual U-net [8]	24,133,013	0	24,133,013

So far, there has been only one study that developed an AI algorithm to detect ascites [3]. In that study, the authors used a CNN algorithm mainly for the classification of three abnormal CT findings of free fluid (ie, ascites), free gas, and mesenteric fat stranding. The accuracy of the CNN algorithm achieved 85% sensitivity and 95% specificity to detect ascites. In contrast, our deep residual U-Net algorithm achieved 96% sensitivity and 96% specificity for ascites detection. In addition, our deep residual U-Net algorithm also quantified the amount of ascites with high segmentation accuracy (mIoU=0.87). Thus, we believe that it is quite possible to use our proposed algorithm for ascites detection and quantification on abdominopelvic CT images in patients who visit the emergency department.

In the majority of urgent and emergent situations, clinicians should read the CT scan without radiologic support immediately after the CT scan was obtained. Getting a radiology report usually takes time, and radiologic support may not be maintained 24 hours per day in many institutions [20]. AI algorithms can help maintain radiology support in real time with high diagnostic accuracy. Our training and test data sets were unique in that CT data were obtained from patients who visited the emergency department of a tertiary care hospital, which is designated as a regional emergency medical center and a regional trauma center in Korea. Currently, we incorporated our deep residual U-Net algorithm in our radiology unit and will start further training of our algorithm in a sustainable manner.

There were false positive cases in which our AI algorithm identified fluid within organs, such as the bladder and ovarian cysts, as ascites (Figure 3). These false positive cases will decrease as we continue to train the AI algorithm. All the false negative cases showed a small amount of ascites, especially between internal organs, such as the bowels, bladder, and uterus. Further training will increase the sensitivity of the AI algorithm to detect ascites.

We adopted a 2D AI algorithm for sequential 2D image analyses rather than a 3D framework, because 3D deep learning requires higher computational power than 2D deep learning [20]. In an emergent clinical setting, a rapid AI algorithm may be preferable to a complex and slow algorithm. Our study showed that sequential 2D image analyses could provide excellent diagnostic accuracy for detecting and quantifying ascites.

### Limitations and Future Work

Our study has several limitations. Firstly, we trained our model using a relatively small amount of CT data. Thus, we will establish a sustainable AI training system and train our AI algorithm using real-world CT data prospectively obtained from our emergency department. Secondly, our AI model was validated internally using a split testing data set. The testing data set was obtained from the same source as the training data set. This may raise issues of generalizability and overfitting of our model [21]. Thus, in the near future, we will validate our model using data from various institutions.

### Conclusions

We propose our deep residual U-Net algorithm for the automatic detection and quantification of ascites in abdominopelvic CT scans. Our model outperformed other state-of-the-art segmentation algorithms based on U-Net, bidirectional U-Net, and R2U-Net.

## Abbreviations

AI: artificial intelligence
AUROC: area under the receiver operating characteristic curve
CNN: convolutional neural network
CT: computed tomography
DICOM: Digital Imaging and Communications in Medicine
DLM: deep learning model
FN: false negative
FP: false positive
HU: Hounsfield Unit
mIoU: mean intersection over union
R2U-Net: recurrent residual U-Net
ROC: receiver operating characteristic
TN: true negative
TP: true positive
